# Prevalence and Sequence-Based Identity of Rumen Fluke in Cattle and Deer in New Caledonia

**DOI:** 10.1371/journal.pone.0152603

**Published:** 2016-04-04

**Authors:** Laura Cauquil, Thomas Hüe, Jean-Claude Hurlin, Gillian Mitchell, Kate Searle, Philip Skuce, Ruth Zadoks

**Affiliations:** 1 Institut Agronomique néo-Calédonien, Connaissance et amélioration des agrosystèmes, Laboratoire de Parasitologie, BP 73, 98890, Païta, Nouvelle Calédonie; 2 Moredun Research Institute, Pentlands Science Park, Penicuik, EH26 0PZ, United Kingdom; 3 Centre for Ecology & Hydrology, Bush Estate, Penicuik, EH26 0QB, United Kingdom; 4 Institute of Biodiversity, Animal Health and Comparative Medicine, College of Medical, Veterinary and Life Sciences, University of Glasgow, Glasgow, G61 1QH, United Kingdom; US Geological Survey, UNITED STATES

## Abstract

An abattoir survey was performed in the French Melanesian archipelago of New Caledonia to determine the prevalence of paramphistomes in cattle and deer and to generate material for molecular typing at species and subspecies level. Prevalence in adult cattle was high at animal level (70% of 387 adult cattle) and batch level (81%). Prevalence was lower in calves at both levels (33% of 484 calves, 51% at batch level). Animals from 2 of 7 deer farms were positive for rumen fluke, with animal-level prevalence of 41.4% (29/70) and 47.1% (33/70), respectively. Using ITS-2 sequencing, 3 species of paramphistomes were identified, i.e. *Calicophoron calicophorum*, *Fischoederius elongatus* and *Orthocoelium streptocoelium*. All three species were detected in cattle as well as deer, suggesting the possibility of rumen fluke transmission between the two host species. Based on heterogeneity in ITS-2 sequences, the *C*. *calicophorum* population comprises two clades, both of which occur in cattle as well as deer. The results suggest two distinct routes of rumen fluke introduction into this area. This approach has wider applicability for investigations of the origin of rumen fluke infections and for the possibility of parasite transmission at the livestock-wildlife interface.

## Introduction

Paramphistomes, or rumen fluke, are the most common parasites in the rumen and reticulum of cattle, buffalo, sheep, deer and other ruminants. They belong to the trematode phylum of helminth parasites and, apart from their organ predilection within the definitive host, they have a life-cycle similar to that of the liver fluke, *Fasciola hepatica*. Adult paramphistomes live on the lining of the first stomach of grazing ruminants and release eggs into the lumen of the gastrointestinal tract, resulting in shedding of eggs in the host’s faeces. Once embryonated, the eggs hatch to release miracidia, which are temporarily free-moving within the environment. Miracidia locate and penetrate a suitable molluscan intermediate host, typically a small snail living in a wet environment. The immature stages develop within the snail host until cercariae are shed and the resultant infectious metacercarial cysts are ingested by the definitive host. The immature paramphistomes penetrate the duodenal mucosa and migrate to the rumen where adult parasites attach to the ruminal surface with their large posterior suckers (acetabula), feeding on ruminal contents. Light infestations and the presence of adult parasites do not appear to cause serious damage or production losses in infected animals [1, 2], but the migration of large numbers of immature paramphistomes through the intestinal mucosa has been shown to cause clinical signs, such as diarrhoea, weight loss, stunting and even death [3, 4, 5].

Rumen fluke have a wide geographic distribution, particularly in tropical and sub-tropical regions of the world, e.g. Australia [6], Thailand [7, 8], Pakistan [9], Mexico [10], Ethiopia [11], and Nigeria [12, 13]. Different species of amphistome or paramphistome dominate in different countries. For example, *Calicophoron calicophorum* is the most common species in Australia whilst *Paramphistomum cervi* is described as the most common species in countries as far apart as Pakistan and Mexico [14, 10, 9, 6]. In Mediterranean and temperate regions of Algeria and Europe, *Calicophoron daubneyi* predominates [15, 1, 16], and it has recently also been recognized as the main rumen fluke in the British Isles [17, 18]. Traditionally, rumen fluke species were identified on the basis of morphology, but microscopic methods are increasingly being replaced with molecular typing methods, including PCR and sequencing approaches targeting the ITS-2 or tRNA-Thr/Cox1 regions [17, 19, 18].

Within a country or region, cattle may or may not harbour the same rumen fluke species as deer. For example, *C*. *daubneyi* was found in cattle but not in roe deer in Galicia, Spain, or in Sika deer in the Republic of Ireland (ROI) [20, 21]. By contrast, fallow deer and red deer in the latter country did have *C*. *daubneyi*, as do cattle. In fallow deer and red deer, *Paramphistomum leydeni* was also detected, but this species has not been reported from cattle in ROI [21, 18]. *P*. *leydeni* has also been detected in deer from Slovakia (Mitchell, Skuce and Zadoks, unpublished data). New Caledonia is a tropical to semi-tropical island in the Southwest Pacific Ocean with considerable cattle and deer populations. Livestock are essentially raised on the main island, distributed over approximately 500 farms. The main island of New Caledonia also has numerous deer (*Cervus timorensis russa*), thought to have been introduced in the 1870s from as few as twelve animals from the island of Java [22]. Some farmers capture wild deer in order to fatten them and slaughter them after 3 months in captivity.

The presence of paramphistomes in cattle and deer has not been investigated in New Caledonia before.

This study presents the results of an abattoir survey to estimate the prevalence of infection in cattle and deer, to determine the species identity of paramphistomes in both host species. In addition, we show how molecular typing at subspecies level can be used to identify potential routes of introduction and transmission of paramphistomes between livestock and wildlife.

## Materials and Methods

### Study Area

New Caledonia, located in the southwest Pacific Ocean, approximately 1,210 km east of Australia, has a tropical to subtropical climate that is strongly moderated by the oceanic influence and the Trade Winds, which attenuate humidity. Relative humidity is around 80%, with a hot and humid period from December to April, temperatures between 27°C and 30°C, and between 100–400 mm of rain per month. This is typically followed by a cooler, drier period from June to August, with temperatures between 20°C and 23°C and between 40–80 mm of rain per month. The two seasons are linked by short transition periods. The mainland of New Caledonia is divided length-wise by a central mountainous region. Most farms are located along the West Coast with beef breeding as their primary activity, particularly extensive breeding of about 100 animals per farm. The majority of animals are Limousin and crosses between Limousin and Brahman or other tick-resistant cattle, such as Droughtmaster, Senepol and Santa Gertrudis. Calves are born year-round and raised with adults until weaning (from 6 to 12 months old), when they are either slaughtered or separated into another herd. The OCEF (Office de Commercialisation et d’Entreposage Frigorifique) slaughterhouse in Bourail is the only cattle slaughterhouse on the island and centralizes processing of the majority of the animals from the territory, including calves and adults. In addition to cattle, deer are fattened on farms, after being captured from the wild. Deer are typically slaughtered at the same official slaughterhouse as the cattle in this study. Permission to sample at the abattoir was granted by the Office de Commercialisation et d’Entreposage Frigorifique.

### Abattoir survey

This study was conducted at the OCEF slaughterhouse, during two periods: May-June 2013 and October-November 2013. No animals were killed specifically for this study and, because the work did not involve live animals, no government approval or licence was required. These periods correspond to the end of the rainy summer and the end of the dry winter, respectively. Cattle belonged to 140 batches and originated from 102 farms, with 36 farms providing adult cattle only, 37 farms providing calves only, and 29 farms providing both adults and calves. A batch was defined as a group of animals belonging to a single age category, originating from a single farm and sent to slaughter on the same date. For example, a farm could send a batch of calves and a batch of adults for slaughter on a single date, or one batch of calves in May-June and one batch of calves in Oct-Nov. In total, 387 adult cattle and 484 calves were inspected. A similar number of deer were inspected (n = 414), albeit from a smaller number of farms, i.e. 324 animals from 5 farms in May and 90 from 2 farms in October. All animals were individually inspected by direct observation of the reticulo-rumen at slaughter. Each rumen was completely opened, emptied and washed prior to inspection. Pink parasites were easily observed on the grey rumen surface. Immature paramphistome stages in the intestine were not sought. A subset of representative adult paramphistomes from cattle and deer, selected on morphological criteria (small, standard, large, thin or stocky) were initially washed with water and then stored in 70% ethanol for species identification with molecular methods. The category (calf/adult cattle/deer) and the provenance of each animal were recorded. The choice of farms and the number of animals inspected per farm depended on the organization of the slaughter schedule and was largely beyond the control of the researchers.

### Statistical Analysis

Data were checked for outliers and missing values and descriptive and univariate analyses were conducted in Statistix 10 (Analytical Software, La Jolla, CA). The observed animal-level prevalence of rumen fluke was calculated for the two age groups (adult vs. calves) and sampling seasons (end of summer vs. end of winter). To determine whether prevalence differed between age groups, seasons and abattoir submissions containing a single age group vs. both age groups, we fitted a generalized linear mixed model including a structural random effect term to account for unmeasured herd level variation, and an individual level random effect term to account for overdispersion. The model assumed a binomial distribution for the number of animals to test positive out of *n* animals tested (animal-level prevalence) per *i*th batch, and was fitted using the ‘lme4’ package in R [23]:

$${\text{prevalence}}_{i}={\text{age}}_{i}+{\text{season}}_{i}+{\text{submission type}}_{i}+\varepsilon_{herd}+i_{od}$$

where age (adult = 1, calf = 0), season (end of summer = 1, end of winter = 0) and submission type (single age group = 1, both age groups = 0) were included as categorical fixed effects, Ɛ_herd_ was a structural random effect at the herd level, and *i*_*od*_ was an individual level random effect accounting for overdispersion. Two-way interactions between main effects were also considered. Significance was declared at *P* < 0.05. To further examine the interaction between season and submission type we also fitted a generalized linear mixed model to calf-only and cow-only data using the model structure outlined above but omitting age as independent variable.

Batch level presence of rumen fluke (binary response variable: 0,1) was analysed using a generalized linear mixed model with a Bernouilli distribution:

$${\text{presence}}_{i}={\text{age}}_{i}+{\text{season}}_{i}+{\text{submission type}}_{i}+\varepsilon_{herd}$$

where age (adult = 1, calf = 0), season (end of summer = 1, end of winter = 0) and submission type (single age group = 1, both age groups = 0) were included as categorical fixed effects, and two-way interactions between main effects were also considered. This model included a structural random effect at the herd level to accommodate unaccounted for variation between herds. Significance was declared at *P* < 0.05.

### Molecular Analysis

Genomic DNA was extracted from individual fluke specimens using the DNEasy Blood and Tissue Kit (QIAGEN, Germany), according to the manufacturer’s recommendations. The ITS-2 region of the rDNA gene, plus the flanking 5.8S and 28S sequences, were amplified by PCR using the generic trematode primers, ITS-2For 5’-TGTGTCGATGAAGAGCGCAG-3’ and ITS-2Rev 5’-TGGTTAGTTTCTTTTCCTCCGC-3’, as described by Itagaki et al. (2003) [24]. PCR was conducted in 25 μl reaction volumes comprising of 1μl genomic DNA template, 12.5 pmol of each primer (Eurofins, Germany), 0.2 mM of each dNTP (Invitrogen, USA), 2 mM MgCl2 and 2.5 U Platinum *Taq* polymerase in 1× Platinum *Taq* buffer (Invitrogen, USA). The PCR was carried out on an Applied Biosystems 2720 PCR machine under the following conditions: 95°C for 10 min; 35 cycles of 94°C for 1 min; 53°C for 1 min; 72°C for 1 min; followed by 72°C for 10 min. PCR products were separated on a 1.2% agarose gel prepared in Tris–acetate–EDTA (TAE) buffer incorporating GelRed (Cambridge Bioscience, UK) and visualised on a UV transilluminator. PCR products of the appropriate size (~500 bp) amplified from individual rumen fluke were purified using QIAquick PCR Purification Kit (QIAGEN, Germany) as specified by the manufacturer. PCR products were eluted using 30μl Elution Buffer and supplied to MWG Eurofins (Germany) at a concentration of 5ng/μl, in 15μl, along with the ITS-2For primer, for direct nucleotide sequencing. The chromatogram files obtained for the forward and reverse sequences were aligned using Lasergene 10 core suite Software SeqMan Pro (DNASTAR, USA) to assess the quality of the sequences and assemble the full-length ITS-2 fragment in order to compare with reference sequences in GenBank using BLASTn at the European Bioinformatics Institute website (http://www.ebi.ac.uk/).

Paramphistomes which were identified as *Calicophoron calicophorum* were further analysed through amplification of the mitochondrial DNA encoding transfer RNA (tRNA-Thr/Cox1) region using the primers described by Martínez-Ibeas et al (2013) [25]: tRNA-ThrFor 5’-TGGAGAGTTTGGCGTCTTTTT-3’ and tRNA-ThrRev 5’-CCATCTTCCACCTCATCTGG-3’. The 25 μl reaction contained 2μl of DNA template, 20 pmol of each primer, 0.2 mM of each dNTP, 2.5 mM MgCl2 and 0.1U Platinum *Taq* polymerase in 1× Platinum *Taq* buffer. The thermocycler was programmed as follows: 92°C for 2 min; 38 cycles of 95°C for 60 secs; 65°C for 30 secs; 72°C for 90 secs; followed by 72°C for 10 min. The amplified product of 885 bp was visualised and sequenced as previously described. Two forward and one reverse sequence for each individual were obtained for alignment using Lasergene 10 core suite software SeqMan Pro (DNASTAR, USA) to provide a consensus. The tRNA-Thr/Cox1 sequences were aligned by MUSCLE using Alignment Explorer in MEGA6 [26] and trimmed to equal lengths. A Neighbour-Joining phylogenetic tree was constructed in Tree Explorer to assess the level of heterogeneity. A published *Calicophoron daubneyi* sequence from the same locus was used as outgroup (Accession number: KJ574046.1, [18]). Tree reliability was assessed by the bootstrap method with 1000 pseudoreplicates.

## Results

### Prevalence and Distribution

**Fig 1:**
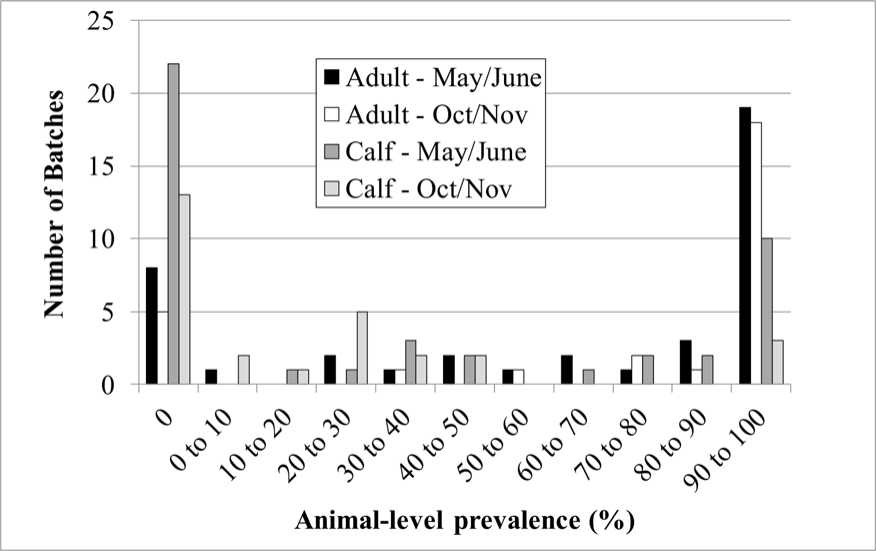
Distribution of animal-level prevalence of paramphistomes in batches of cattle at slaughter by age group and observation period. Animal-level prevalence is shown in categories, where by the first value of the range is excluded and the last value is included. Negative batches are shown as a separate category at animal-level prevalence of zero.

The number of animals inspected per herd ranged from 1 to 25 for cattle (calves or adult) and from 39 to 74 for deer. Cattle herds were located in 16 of the 33 municipalities of the island, with 85% of the herds inspected located on the West coast. These municipalities largely correspond to the main cattle breeding area of New Caledonia. Between 1 and 17 herds were surveyed per municipality. The number of batches per farm was 1, 2 or 4 for 68, 64 and 8 farms, respectively, with 4 batches being the highest number possible (both age groups in both seasons). Prevalence of paramphistomes was higher for adult cattle than for calves, both at batch level and at animal level (Tables 1 and 2). More than half of adult batches (40 of 70 or 57%) had animal-level prevalence higher than 90%, whereas more than half of the calf batches were not infected (37 of 72 or 51%). By contrast, only 19% of adult batches were not infected, and only 18% of calf batches (13 of 72) had animal-level prevalence higher than 90%. Thus, adult batches were more likely to be infected and, if they were infected with paramphistomes, most of the animals were positive, whereas calf batches were less likely to be infected and within infected calf batches, relatively few animals were positive (Fig 1). Two of 7 inspected deer farms had paramphistomes, with intra-herd prevalence of 41.4% (29/70) and 47.1% (33/70), respectively.

**Table 1:** Animal-level prevalence for cattle slaughtered in New Caledonia in 2013.

Age	Season	Age groups	Positive/Tested	Prevalence (95% Confidence Interval)
Adult	May/June	Single	88/161	54.7% (46.9 to 62.2)
Adult	Oct/Nov	Single	92/110	83.6% (75.6 to 89.4)
Adult	May/June	Both	66/86	76.7% (66.8 to 84.4)
Adult	Oct/Nov	Both	24/30	80.0% (62.7 to 90.5)
Calf	May/June	Single	39/162	24.1% (18.1 to 31.2)
Calf	Oct/Nov	Single	27/124	21.8% (15.4 to 29.8)
Calf	May/June	Both	79/123	64.2% (55.4 to 72.1)
Calf	Oct/Nov	Both	16/75	21.3% (13.6 to 31.9)

Proportion of animals testing positive for rumen fluke by age group, season and type of submission (single age group vs. both age groups) submitted by the same farm on the same date.

**Table 2:** Batch-level prevalence for cattle slaughtered in New Caledonia in 2013.

Age	Season	Type of submission	Positive/Tested	Prevalence (95% Confidence Interval)
Adult	May/June	Single	14/19	73.7% (51.2 to 88.2)
Adult	Oct/Nov	Single	16/18	88.9% (67.2 to 96.9)
Adult	May/June	Both	18/21	85.7% (65.4 to 95.0)
Adult	Oct/Nov	Both	7/10	70.0% (39.7 to 89.2)
Calf	May/June	Single	7/24	29.2% (14.9 to 49.2)
Calf	Oct/Nov	Single	9/17	52.9% (31.0 to 73.8)
Calf	May/June	Both	15/20	75.0% (53.1 to 88.8)
Calf	Oct/Nov	Both	6/11	54.5% (28.0 to 78.7)

Proportion of batches testing positive for rumen fluke by age group, season and type of submission (single age group vs. both age groups submitted) by the same farm on the same date. A batch is defined as a group of animals of the same age (calf vs. adult) sent to slaughter from the same farm on the same date. Some farms submitted animals from a single age group (i.e. a batch of calves or a batch of adults) whereas other farms submitted animals from both age groups (i.e. a batch of calves as well as a batch of adults).

**Fig 2:**
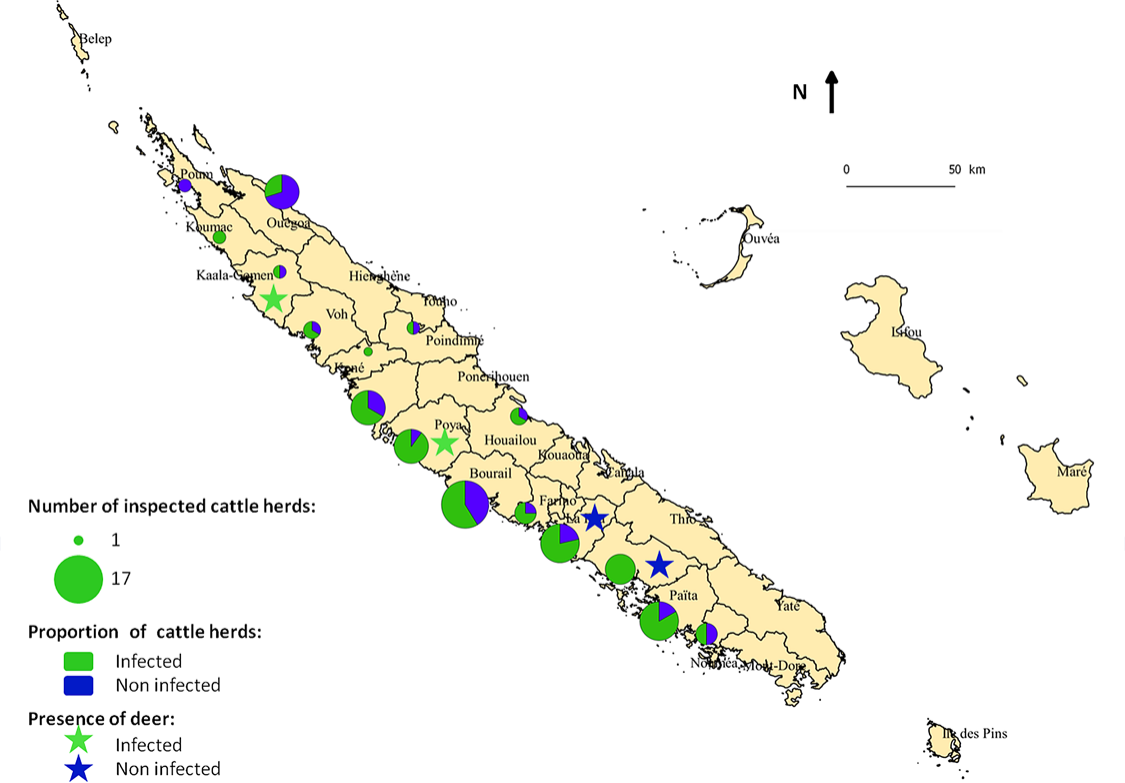
Geographical distribution of inspected cattle and deer herds in New Caledonia. Pie charts indicate the inter-herd prevalence of paramphistomes in cattle for each municipality, where the size of the pie chart is proportional to the number of herds inspected from that municipality. Stars indicate municipalities with deer and colour indicates whether or not paramphistomes were observed in deer from that municipality.

The geographical distribution of the herds inspected is shown in Fig 2, including the proportion of infected herds per municipality and the detection of paramphistomes in deer. With the exception of one municipality, Poum, at the north-western tip of the main island, paramphistomes were found in cattle from every municipality. The proportion of infected herds varied from 0 to 100%. The mean percentage of infected herds per municipality was 68% (SE = 27%). Among deer farms, paramphistomes were detected in the North-western half of the island but not in the South-eastern half, whereas no obvious association between region and prevalence could be observed in cattle farms.

For animal-level prevalence, when only main effects were considered, prevalence was significantly correlated with age and submission type but not with season (Table 3). Mixed batches (calves with cows) had significantly higher prevalence than single batches (only calves or only cows) and calves had significantly lower prevalence than cows but no difference in prevalence between batches tested later or earlier in the season was detected. Including interactions between the three main effects demonstrated significant dependence amongst the three variables. The effect of age was significantly different between submission types and seasons, whilst the effect of submission type was also significantly different between seasons (Table 4). Inspection of Table 1 suggested that interactions between independent variables might differ between age groups. To explore this, calf and cow data were analysed separately. For animal-level prevalence in calves, the interaction between submission type and season was significant (P = 0.02), but for cows it was not (P = 0.10), confirming the complex interaction between independent variables.

**Table 3:** Binomial general linear mixed model animal-level data output (i).

Fixed effect	Estimate	Standard error	Z-value	P-value
Intercept	0.8641	0.7580	1.140	0.25
Age	-4.1228	0.8863	-4.651	<0.001
Submission type	1.922	0.9275	2.073	0.04
Season	1.0296	0.8431	1.221	0.22

Estimates for the binomial general linear mixed model fitted to all animal-level data with main effects for age, submission type and season, with a structural herd level random effect.

**Table 4:** Binomial general linear mixed model animal-level data output (ii).

Fixed effect	Estimate	Standard error	Z-value	P-value
Intercept	0.2746	0.9066	0.303	0.76
Age	-4.3655	1.3172	-3.314	<0.001
Submission type	2.0700	1.2555	1.649	0.10
Season	3.9682	1.2543	3.164	0.002
Age*Submission type	2.7100	1.3625	1.989	0.047
Age*Season	-2.5307	1.2758	-1.984	0.047
Submission type*Season	-4.2555	1.5966	-2.665	0.008

Estimates for the binomial general linear mixed model fitted to all animal-level data with main effects for season, submission type and age, and 2-way interactions, with a structural herd level random effect.

At batch-level, when only main effects were considered, presence of rumen fluke was significantly correlated with age, but not with submission type or season (Table 5). As for animal-level data, cows had significantly higher probability of having rumen fluke than calves. Although submission type and season were not significant as main effects, the interaction between submission type and season was significant (Table 6). There was no significant interaction between age and the other main effects.

**Table 5:** Binomial general linear mixed model rumen fluke-level data output (i).

Fixed effect	Estimate	Standard error	Z-value	P-value
Intercept	2.8533	0.9408	3.033	0.002
Age	-1.6627	0.5538	-3.002	0.003
Submission type	0.9372	0.5486	1.708	0.088
Season	0.4648	0.5617	0.828	0.41

Estimates for the binomial general linear mixed model fitted to rumen fluke batch-level data with main effects for season, submission type and age, with a structural herd level random effect.

**Table 6:** Binomial general linear mixed model rumen fluke-level data output (ii).

Fixed effect	Estimate	Standard error	Z-value	P-value
Intercept	3.59478	1.47586	2.436	0.01
Age	-2.35487	0.92407	-2.548	0.01
Submission type	-0.67222	1.60422	-0.419	0.67
Season	1.22454	1.66339	0.736	0.46
Age*Submission type	1.52139	0.99130	1.535	0.12
Age*Season	0.03738	0.93547	0.040	0.97
Submission type*Season	-2.18071	1.01697	-2.144	0.03

Estimates for the binomial general linear mixed model fitted to rumen fluke batch-level data with main effects for season, submission type and age, interactions between main effects, and a structural herd level random effect.

### Molecular Characterization

Amplification of the ITS-2 region fragments ranging from 350 to 455 bp. For sequence analysis, fragments were trimmed to 344 bp Most fragments contained the entire ITS-2 region (282bp) plus the flanking 5.8S and 28S sequences. Among 79 fragments that were successfully sequenced, comprising 54 from cattle and 25 from deer, BLAST analysis revealed the presence of *C*. *calicophoron* (n = 57), *Fischoederius elongatus* (n = 12) and *Orthocoelium streptocoelium* (n = 10). All sequences were between 99.3–100% homologous to *C*. *calicophoron*, *F*. *elongatus* and *O*. *streptocoelium* sequences (accession numbers: GU133057, GU133062 and GU133058, respectively). All three species were detected in cattle as well as deer, although *C*. *calicophoron* dominated in cattle (Table 7). Detailed analysis of prevalence within host species is not meaningful because selection of specimens for species identification was not randomized but aimed at maximising phenotypic differences among specimens. The three species show inter-specific variation in the ITS-2 region, whereby *F*. *elongatus* and *O*. *streptocoelium* showed 10 and 14 SNPs, respectively, when compared to *C*. *calicophoron* across a 149bp sequence. All SNPs were single base substitutions.

**Table 7:** Species identity of individual rumen fluke specimens by host species, as revealed by ITS-2 sequence analysis.

Rumen fluke species	Host species		Total
	Cattle	Deer	
*Calicophoron calicophorum*	46	11	57
*Fischoederius elongatus*	5	7	12
*Orthocoelium streptocoelium*	3	7	10
Total	54	25	79

**Fig 3:**
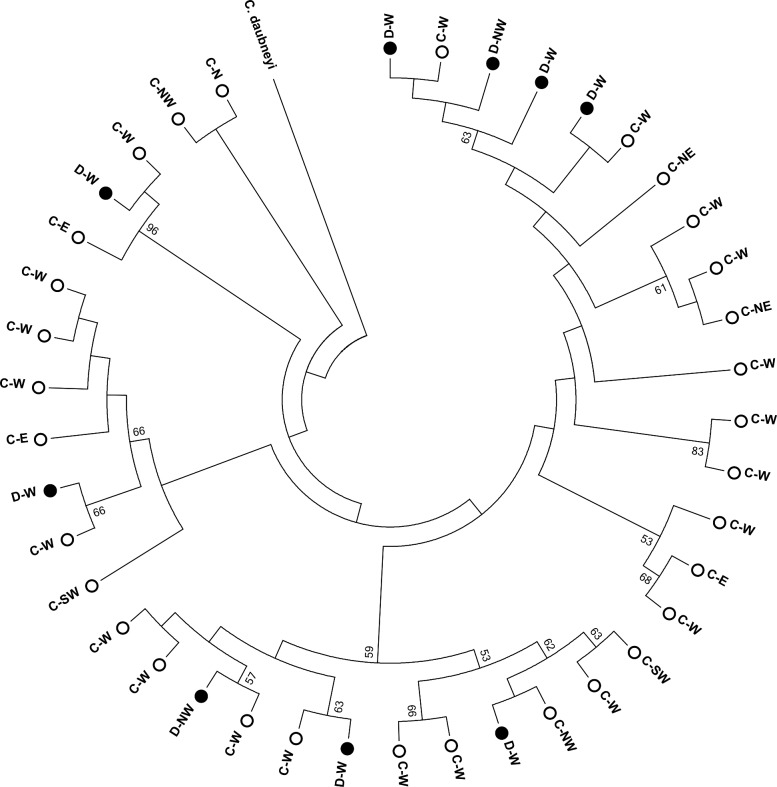
Phylogenetic tree showing the topological relationship between mtDNA rRNA-Thr/Cox1 fragments of *Calicophoron calicophorum* from cattle (white dots, C) and deer (black dots, D) using *Calicophoron daubneyi* as outgroup. Numbers along branches indicate bootstrap support (values over 50 shown). Where available, data on region of origin is included (N = North; NE = North-East; E = East; SW = South West; W = West; NW = North West).

The mtDNA tRNA-Thr/Cox1 primers successfully amplified the desired region from 40 *C*. *calicophorum* individuals with products ranging from 718–821 bp in length. After alignment, these were trimmed to a consistent length of 678 bp before creating the phylogenetic tree. There was heterogeneity within the *C*. *calicophorum* sequences, but there was no clustering by region or host species (Fig 3). Typing of *F*. *elongatus* and *O*. *streptocoelium* at subspecies level was not successful, i.e. no amplicons were obtained with the method that was used for *C*. *calicophorum*.

## Discussion

Our study showed that paramphistomes are widespread among the cattle and deer populations of New Caledonia. Few studies exist on national prevalence in any given country, as there is typically no active surveillance for rumen fluke [27]. Instead, prevalence reports tend to represent regional data, sometimes from a limited number of animals and without clear description of their epidemiological origin. The choice of farms included in the present study was not random either but was dictated by the slaughterhouse schedule. However, our sample set did reflect the distribution of cattle farms on New Caledonia. Indeed, 92.9% of livestock are located in the West of the island (from Païta to Poum) and 43.1% are in Bourail, La Foa and Païta municipalities (unpublished data from Chambre d’Agriculture de Nouvelle-Calédonie), which corresponds to 42.6% of the farms sampled. To our knowledge, the only previous prevalence study in the Pacific area was conducted in cattle in Queensland, Australia and revealed a prevalence of 12.5% in cattle [28], which is much lower than the prevalence observed in New Caledonia.

In our survey, the intra-herd prevalence for adult cattle was much higher than in calves. This observation could potentially be explained by accumulation of paramphistomes over time. The longevity of paramphistome parasites would allow them to remain within the definitive host for a long period, with intra-host survival of several years reported for some rumen fluke species, e.g. *Paramphistomum microbothrium* [29, 30]. Specific information on longevity for the rumen fluke species detected in our study is not available. Given that rumen fluke is not considered a clinical or production problem in New Caledonia, treatment targeting this parasite is highly unlikely. Moreover, it is also unlikely that the rumen fluke will have been successfully removed as a by-product of treatment for liver fluke because, unlike many other regions where rumen fluke is endemic, the island is liver fluke-free [31]. In the absence of treatment, animals can remain infected for a long time and they may provide continual contamination of the pasture with paramphistome eggs, thus contributing to continuation of the life cycle, if appropriate intermediate host snails are present. In adult cattle, prevalence had a more or less bimodal “all or nothing” distribution (Fig 1), suggesting that farms are either positive or negative for rumen fluke and that all cattle on positive farms are exposed to rumen fluke over the course of their life. There was no obvious association between rumen fluke presence and geographic location (Fig 2). Other herd level risk factors that may explain this distribution were not explored in the current study. In calves, prevalence was lower, as might be expected based on shorter life span and hence shorter exposure time [27]. In May-June, the prevalence of rumen fluke in calves largely showed an “all or nothing” distribution similar to that for adult cattle. In October-November, however, a within-batch prevalence of 20–30% was also commonly observed for calves, implying that exposure and infection levels are starting to build at that time of year. Season was not significantly associated with animal-level prevalence when only main effects were included in the model, but it was significant when interactions were considered (Tables 3 and 4). Specifically, the observed animal-level prevalence in calves was higher in mixed submission from May/June than in any other category of calves (Table 1). It is unclear whether this is a biological phenomenon or due to a type I error. Similar difficulties in analysis of risk factors for rumen fluke presence at slaughter were encountered in a study from the Republic of Ireland [27].

There was no obvious clustering of positive herds by municipality, a phenomenon similar to the wide range of liver fluke prevalences observed within some post-code regions in the UK [32]. The geographic distribution of infected herds would be expected to be linked with the distribution of the intermediate host snail. Franc (1953) [33] made an inventory of fluvial and terrestrial molluscs in New Caledonia, highlighting the presence of representatives of the genera, *Planorbis* and *Bulinus*, both known to be potential intermediate hosts for some species of paramphistomes in certain parts of the world [34]. Rolfe *et al*. (1991) [6] described the epidemiology of paramphistomosis in cattle in Australia. Peak fluke burdens coincided with prolonged rainfall and inundation of grazing areas, resulting in rapid multiplication and infection of host snails, specifically, *Gyraulus scottianus* and *Helicorbis australiensis*. However, we do not know which snail is host to paramphistomes in New Caledonia, or which environmental and climatic conditions are required for this species.

Although the identity of intermediate host snail species remains unknown, the current study revealed that rumen fluke of cattle and deer belong to three species. In cattle, the dominant species was *C*. *calicophorum*. This species has previously been reported in New Caledonia based on morphological identification [6] and its presence was confirmed here using modern molecular methods. It is also the main paramphistome species found in cattle in Australia and New Zealand [14]. The proximity of these territories and their respective trade routes could allow for some exchange of livestock and livestock parasites. *Paramphistomum ichikawai* and *Ceylonocotyle streptocoelium* are also present in Australia [14], but were not found during our study. The other rumen fluke species identified in the present survey, *F*. *elongatus* and *O*. *streptocoelium*, would appear to be of oriental origin. *F*. *elongatus* is known to be present in Thailand [8], The Philippines [35], India [36] and Indonesia [37]. Presence of *O*. *streptocoelium* has been reported from Thailand (Sripalwit *et al*., 2007), Japan (Itagaki *et al*., 2003) and India [38]. The presence of these rumen fluke species in New Caledonia could be a result of the introduction of Java deer (*Cervus timorensis russa*) from Indonesia in the 1870s [22]. Abattoir inspection of 4,000 farmed deer from across the island between 1987 and 1991 identified occasional lungworm (*Dictyocaulus viviparus*) infections, but it is not clear whether the rumen was inspected for paramphistomes [22]. In the west of the island, both deer and cattle are abundant, and co-grazing is common. Thus, cross-infection between cattle and deer is possible, which may explain why all three rumen fluke species identified in this study were found in both host species, in contrast to the situation in countries were co-grazing is not as common, e.g. Spain and ROI [20, 21]. Further evidence for cross-infection between cattle and deer in our study is provided by sub-genotyping of *C*. *calicophorum*, which did not provide any indication of clustering of subtypes by host species. Thus, although the route of introduction may have differed between the three fluke species, with *C*. *calicophorum* most likely introduced via cattle from Australia or New Zealand and *F*. *elongatus* and *O*. *streptocoelium* possibly introduced via deer from Indonesia, there is currently no clear boundary between the host species.

## Conclusions

This study highlighted the high prevalence of paramphistomes in New Caledonian cattle with infection detected in approximately two-thirds of the inspected herds. Prevalence in calves was higher during the wet and humid season, from March to April, than during the dry season from October to November. Both in cattle and in deer, three rumen fluke species were identified based on ITS2-sequencing, viz. *C*. *calicophorum*, *F*. *elongatus* and *O*. *streptocoelium*, suggesting that rumen fluke species may be transmitted between host species. Within *C*. *calicophoron*, sequencing of the tRNA-Thr/Cox1 region provided differentiation at subspecies level and highly similar to identical sequences were found across the two host species, lending further support to the possibility of inter-species transmission. The molecular typing methods described here are useful to study the potential for rumen fluke transmission at the livestock-wildlife interface, although further methods development is needed for subspecies typing of *Fischeroedius* and *Orthocoelium* species. For detailed understanding of the mechanism of inter-species transmission and of risk factors that may explain differences in within-herd prevalence among cattle and deer herds, further epidemiological studies are needed. Such studies should consider herd management as well as environmental risk factors and the role of intermediate host snails.
